# Taxonomic diversity of microbial communities in sub-seafloor hydrothermal sediments of the active Santorini-Kolumbo volcanic field

**DOI:** 10.3389/fmicb.2023.1188544

**Published:** 2023-06-29

**Authors:** Paraskevi N. Polymenakou, Paraskevi Nomikou, Mark Hannington, Sven Petersen, Stephanos P. Kilias, Thekla I. Anastasiou, Vasiliki Papadimitriou, Eleutheria Zaka, Jon Bent Kristoffersen, Danai Lampridou, Sandra Wind, Verena Heinath, Sabine Lange, Antonios Magoulas

**Affiliations:** ^1^Hellenic Centre for Marine Research, Institute of Marine Biology, Biotechnology, and Aquaculture, Heraklion, Greece; ^2^Faculty of Geology and Geoenvironment, National and Kapodistrian University of Athens, Athens, Greece; ^3^GEOMAR Helmholtz Centre for Ocean Research Kiel, Kiel, Germany; ^4^Department of Biology, University of Crete, Heraklion, Greece; ^5^Department of Earth and Environmental Sciences, University of Ottawa, Ottawa, ON, Canada; ^6^Institute of Geosciences, University of Kiel (CAU), Kiel, Germany

**Keywords:** submarine volcano, Santorini (Greece), microbial community composition and diversity, diversity, hydrothermal vent field, Kolumbo volcano

## Abstract

**Introduction:**

Active hydrothermal vents of volcanic origin provide a remarkable manifestation of life on Earth under extreme conditions, which may have consequences for our understanding of habitability on other terrestrial bodies as well.

**Methods:**

Here, we performed for the first time Illumina sequencing of bacterial and archaeal communities on sub-seafloor samples collected from the Santorini-Kolumbo volcanic field. A total of 19 (3-m long) gravity corers were collected and processed for microbial community analysis.

**Results:**

From a total of 6,46,671 produced V4 sequences for all samples, a total of 10,496 different Operational Taxonomic Units (OTUs) were identified that were assigned to 40 bacterial and 9 archaeal phyla and 14 candidate divisions. On average, the most abundant phyla in all samples were Chloroflexi (Chloroflexota) (24.62%), followed by Proteobacteria (Pseudomonadota) (11.29%), Firmicutes (Bacillota) (10.73%), Crenarchaeota (Thermoproteota) (8.55%), and Acidobacteria (Acidobacteriota) (8.07%). At the genus level, a total of 286 known genera and candidate genera were mostly dominated by members of *Bacillus, Thermoflexus, Desulfatiglans, Pseudoalteromonas*, and *Pseudomonas*.

**Discussion:**

In most of the stations, the Chao1 values at the deeper layers were comparable to the surface sediment samples denoting the high diversity in the subsurface of these ecosystems. Heatmap analysis based on the 100 most abundant OTUs, grouped the sampling stations according to their geographical location, placing together the two hottest stations (up to 99°C). This result indicates that this specific area within the active Kolumbo crater create a distinct niche, where microorganisms with adaptation strategies to withstand heat stresses can thrive, such as the endospore-forming Firmicutes.

## 1. Introduction

Beneath the sediment surface lies a world of microscopic life inhabited by a great diversity of bacteria and archaea, most of them largely unknown (Jørgensen and Marshall, 2016). Today, it has been estimated that the sub-seafloor microorganisms account for about 2.9 × 10^29^ cells, which is equivalent to half of the microbial cells worldwide in the oceans (Kallmeyer et al., 2012). These extreme sub-seafloor organisms were the first representatives of life on Earth and they are responsible for the genesis of geological structures during the evolution and creation of all currently known ecosystems (Pikuta et al., 2007). So far, most of sub-seafloor studies of microbial life have been mainly performed in environments with *in situ* temperatures < 30°C and thus our knowledge about the habitability of higher temperature environments, such as the hydrothermal vent systems, is still limited (LaRowe et al., 2017). Only recently, Heuer et al. (2020) demonstrated for the first time, the dependence of microbial abundance and activity to critical temperatures around 40 to 50°C and 70°C and showed that life in the deep subseafloor is not constrained by an upper temperature limit below 120°C (Heuer et al., 2020). The geochemical conditions in such systems are favorable for microbial metabolism and they can support colonization of non-photosynthetic, lithotrophic communities (Orcutt et al., 2011; Ivarsson et al., 2013). Microbes isolated from hydrothermal vent environments can live at temperatures up to ?122°C (Takai et al., 2008). These hyperthermophiles are fueled by high fluxes of oxidants and reductants (Heuer et al., 2020). Interestingly, hot hydrothermal vents in some places are dominated by archaea (e.g., Lost City Hydrothermal Field of Mid-Atlantic Ridge, a deep-sea hydrothermal field in the Central Indian Ridge; Takai et al., 2004; Brazelton et al., 2010; Lagostina et al., 2021) whereas in other places by bacteria (Takai et al., 2006; Lagostina et al., 2021). A possible explanation for these differences is that archaea can live in low-energy environments whereas bacteria can cope better in energy-rich or unstable environments (Valentine, 2007; Lagostina et al., 2021).

The Hellenic Volcanic Arc (HVA) located in the Aegean Sea contains unique hydrothermal vents where its development is a response to the subduction of the African plate beneath the active margin of the European plate (Le Pichon and Angelier, 1979; Nomikou et al., 2012). HVA consists of five islands i.e., Methana, Milos, Santorini, Nisiros, and Kos. Among these, the Santorini volcano is world famous because of its recent explosive eruption (∼3,600 years ago) which was one of the largest known volcanic events in historical time. The Santorini volcanic field consists of more than 20 submarine cones with the largest one, the Kolumbo volcano, being located 505 m below sea level. High (up to 220°C) and low (up to 70°C) temperature polymetallic chimneys and vents covers the seafloor at the norther part of the Kolumbo crater. The exterior of the chimneys and large areas of the seabed around Kolumbo were covered with reddish/orange microbial mats and streams of white/gray mats. Major elements in the area include Fe, S, Pb, Na, As, Sb, Mn and Sr with average concentrations of 111,333 ppm Fe, 23,850 ppm S, 6,043 ppm Pb, 4,182 ppm Na, 2,656 ppm As, 2,616 ppm Sb, 2,075 ppm Mn and 1,828 ppm Sr (Christakis et al., 2018). The discharge of gaseous CO_2_ (> 99%) of active vent chimneys of Kolumbo (Rizzo et al., 2016) causes an increase of water density that leads to the accumulation of acidic seawater (as low as pH 5.0) near the crater floor (Carey et al., 2013).

Previous bio-geochemical (Kilias et al., 2013; Christakis et al., 2018) and metagenomic investigation (Oulas et al., 2016) conducted on microbial mat samples covering the seafloor of the Santorini-Kolumbo volcanic system and the surfaces of Kolumbo chimneys, revealed that both Kolumbo crater and Santorini caldera harbor highly complex bacterial and archaeal communities mostly dominated by chemolithoautotrophs, methanotrophs, as well as heterotrophs that perform anaerobic degradation of aromatic hydrocarbons. Kilias et al. (2013) demonstrated that iron microbial-mat in Kolumbo volcano is dominated by ferrihydrite-type phases and microbial sequences akin to “*Nitrosopumilus maritimus*,” a mesophilic Thaumarchaeota strain capable of chemoautotrophic growth on hydrothermal ammonia and CO_2_. This result was further confirmed by Christakis et al. (2018), for microbial communities inhabiting the surfaces of the inactive polymetallic chimneys of Kolumbo volcano. In this study, the active chimney communities were found to be dominated by operational taxonomic units (OTUs) related to thermophilic members of Epsilonproteobacteria, Aquificae, and Deltaproteobacteria whereas the inactive chimney communities were dominated by an OTU closely related to the archaeon *Nitrosopumilus* sp., and by members of Gammaproteobacteria, Deltaproteobacteria, Planctomycetes, and Bacteroidetes. These lineages are closely related to phylotypes typically involved in iron, sulfur, nitrogen, hydrogen and methane cycling.

Although we have demonstrated the enormous structural and functional diversity of seafloor microbial communities in Santorini-Kolumbo volcanic complex, we still do not know anything on the status of its sub-seafloor ecosystems. Of all the potentially habitable environments on Earth, the submarine volcanoes with active hydrothermal vents are among the most intriguing, extreme, and challenging environments to investigate the origins and importance of sub-seafloor communities, the development and maintenance of life and to uncover its valuable genetic resources. The active Santorini-Kolumbo volcanic complex serves as the best candidate to face these quite exciting and cutting-edge research challenges. In the present study, for the first time, microbiological data from sub-seafloor hydrothermal sediments from this unique submarine volcanic-arc setting are presented.

## 2. Results

### 2.1. Lithology and physicochemical characteristics

**FIGURE 1:**
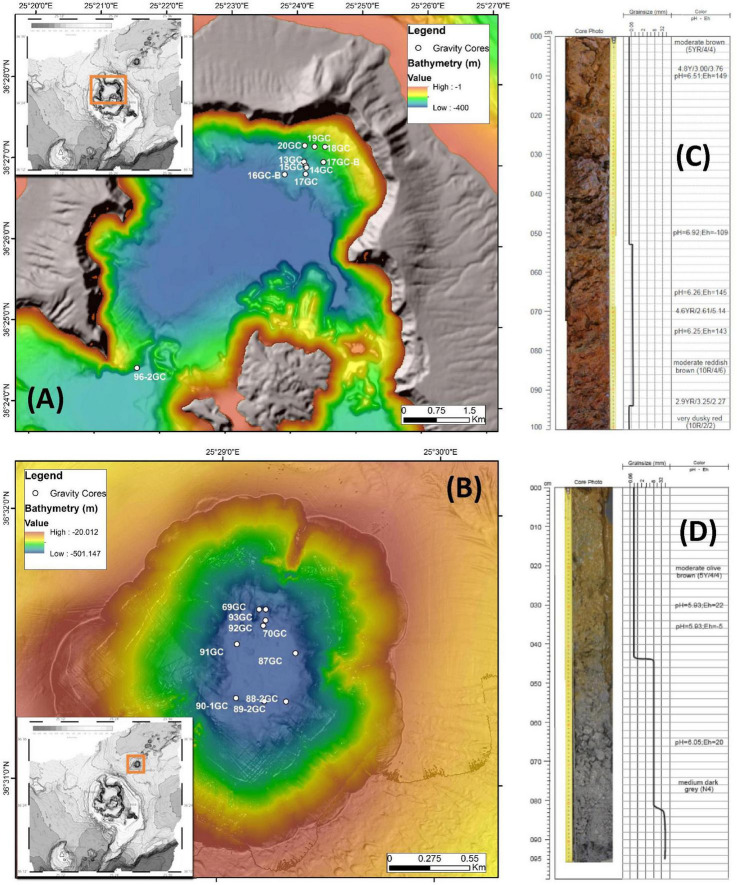
Detailed bathymetry maps showing the locations of gravity coring stations during POS510 expedition in **(A)** Santorini caldera and **(B)** Kolumbo volcano. Top 100 cm of two representative gravity corers of **(C)** Santorini caldera: 20GC and **(D)** Kolumbo volcano: 92GC. Values of pH and Eh of the different layers are also shown.

**FIGURE 2:**
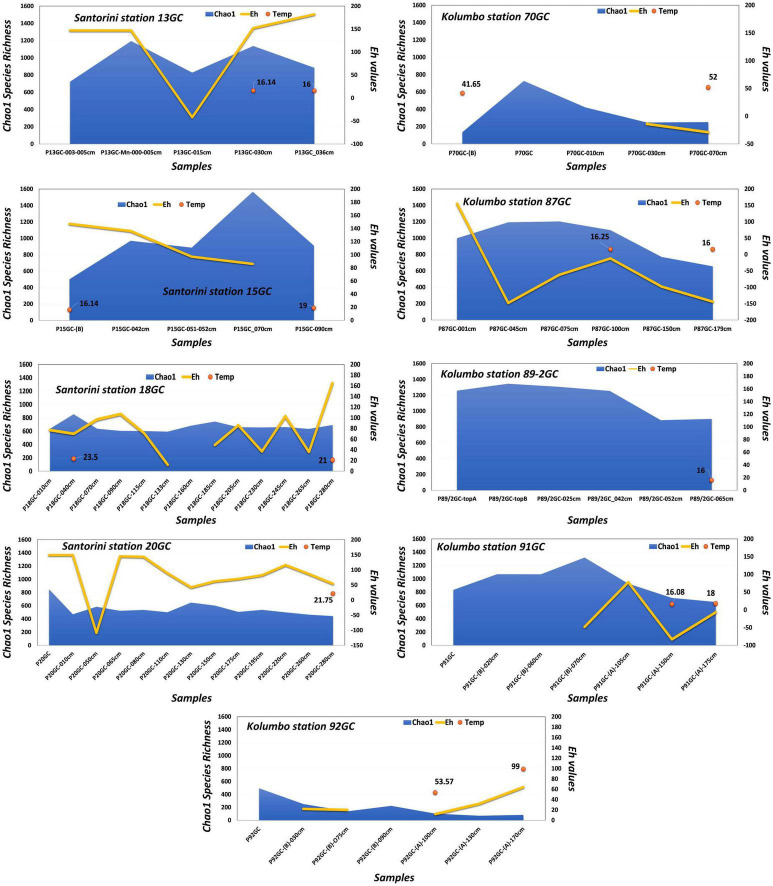
Variation of Chao1, Eh and temperature values of the nine sliced cores.

Stations with numbers from 13GC to 20GC were located at the norther part of Santorini caldera whereas stations numbering from 69GC to 93GC were located inside Kolumbo volcano. Station 96-2GC was located at the southern part of Santorini caldera (Figure 1 and Supplementary Table 1). Core sediments were primarily Fe-oxide rich mud silt and/or Fe-oxide rich reddish-brown silt with Mn or Fe-Mn crusts. Of particular note, several olive brown or olive gray clay zones were observed at the deeper sediment layers in some cores (Supplementary Table 1). Maximum probe temperatures ranged from 15.82°C at station 89-2GC to 53.57°C at station 92GC, both located inside the Kolumbo volcano (Figure 2 and Supplementary Table 2). Probe temperatures inside the Santorini caldera varied between 15.88°C at the southern part of the caldera to 23.5°C at the north. Temperature at the bottom of each corer ranged from 15°C at station 69GC to 99°C at station 92GC, both located inside the crater of Kolumbo volcano. Bottom temperature at Santorini caldera stations varied between 15 and 21°C (Figure 2 and Supplementary Table 2). The minimum pH value was recorded inside the Santorini caldera at the bottom gravity corer of station 20GC (i.e., 5.83), whereas the maximum value was recorded inside the Kolumbo volcano at station 13GC and the sediment layer of 30 cm (i.e., 7.69). Values of redox potential varied from −148 to 182 mV with minimum values close to anaerobic conditions being recorded inside the Kolumbo volcano (Figure 2 and Supplementary Table 1).

### 2.2. Sequencing data analysis

For studying the microbial diversity within each sample, we used direct counts of the number of operational taxonomic units (OTUs) at 97% similarity level, and various metrics that take into account the abundance of the more rare taxa (Chao1), the abundance and distribution of the taxa [Shannon–Weaver diversity index (H) and evenness (E_H_)] and the richness and evenness of the taxa (Simpson D, 1-D, and 1/D indices) (Figure 2 and Table 1). Although, the amplicon sequencing variants (ASVs) have been proposed as an alternative to OTUs for microbial communities analysis, in order our results to be comparable to previous studies at the same investigated area, we decided to follow the classical OTU approach. In addition, according to Schloss (2021), ASVs may artificially split bacterial genomes into separate clusters. Thus, the use of ASVs may lead to conflicting inferences about the ecology of different ASVs derived from the same genome. In the present study, OTUs were defined at 97% sequence similarity, although this threshold may cluster different bacterial species into the same OTU. Results of sequencing analysis allowed the identification of thousands of OTUs for each sample. After processing, a total of 660,252 high quality sequences remained across all 78 samples. Following the recommendations of Salter et al. (2014) and Sheik et al. (2018), we further removed a total of 13,579 sequences assigned to previously identified genera as contaminants from the DNA extraction reagents. A total of 35 genera were removed (Supplementary Table 3), whereas the sequences of the genera *Pseudomonas* and *Bacillus* were kept to our database since our recent investigations have showed them to be among the key players of the Kolumbo volcano (Mandalakis et al., 2019; Bravakos et al., 2021). In addition, we have recently isolated a series of *Bacillus* strains from sample P92GC capable to grow at high temperatures of up to 99°C (unpublished data). The number of retained sequences per sample varied from 2,365 to 18,651. A total of 10,496 OTUs were generated after clustering of the remaining 646,671 sequences at 97% similarity level. The number of OTUs per sample was ranging from 53 to 1,395 (Table 1). Slightly higher OTUs were recorded at Kolumbo stations and at all depths compared to Santorini samples (6,920 and 6,314, respectively; Table 1). Maximum OTU values were calculated at samples P15GC-070cm (1,395; Table 1) and P17GC (1,151; Table 1) of Santorini caldera and at samples P91GC-(B)-070cm (1,148; Table 1) and P90-1GC (1,147; Table 1) of Kolumbo volcano. Interestingly, the minimum values of OTUs were recorded at the deeper sediment layers of station 92GC and more specifically at 100, 130 and 170 cm sediment layers (70, 53, 64, respectively; Table 1) and at the deeper sediment layers of station 70GC (194 and 155 at sediment layers of 30 and 70 cm, respectively). Station 92GC and its adjacent station 70GC were characterized by the higher recorded bottom temperatures of 99 and 54°C, respectively, compared to the rest sampling locations (15–21°C). Chao1, Shannon–Weaver (H) and Simpson Reciprocal (1/D) indices revealed comparable species diversity between Santorini (Chao1 = 6,343; *H* = 5.58; 1/D = 52.33; Table 1) and Kolumbo samples (Chao1 = 6,938; *H* = 6.06; 1/D = 68.66; Table 1). Regarding the Santorini samples, all calculated indices varied from 442 to 1,567 (Chao1), from 2.76 to 6.01 (H) and from 2.85 to 160.46 (1/D) whereas in Kolumbo samples the corresponding values range were 70–1, 1,382 (Chao1), 1.72–5.94 (H) and 3.16–204.48 (1/D). Chao1 reached its maximum value at sample P15GC_070cm of Santorini caldera and at sample P90-1GC, whereas the minimum Chao1 values were calculated at the deeper layers of station 92GC sample [P92GC-(A)-130cm: 70; P92GC-(A)-170cm: 83; Table 1]. Maximum values of the rest diversity indices were recorded at sample P15GC-cc of Santorini seafloor (*H* = 6.01; 1/D = 125.32; Table 1) and at sample P87GC_075cm of Kolumbo seafloor (*H* = 5.94; 1/D = 161.79; Table 1). Minimum values were calculated at the surface layer of Kolumbo sample P70GC-(B) (*H* = 1.91; 1/D = 3.46; Table 1) and at the deeper layer of station 92GC, similarly to the Chao1 values [sample P92GC-(A)-170cm: *H* = 1.72; 1/D = 3.16; Table 1]. Both Kolumbo stations 70GC and 92GC displayed the minimum values of Chao1 whereas in Santorini caldera, the lowest Chao1 values were calculated at stations 18GC and 20GC compared to the rest ones (Figure 2).

**TABLE 1:** Values of OTUs and biodiversity indices (alpha diversity) including Chao1, Shannon–Weaver diversity index (H) and evenness (E_H_) and Simpson Diversity index (D, 1-D, 1/D) for 16S amplicon data.

Samples	Obs. OTUs	Alpha diversity					
		Chao1	Shannon–Weaver (H)	Evenness (E_H_)	Simpson (D)	1-D	1/D
P13GC-003-005cm	580	723	2.76	0.43	0.35	0.65	2.85
P13GC-Mn-000-005cm	981	1,194	5.30	0.77	0.02	0.98	51.44
P13GC-015cm	701	828	5.73	0.88	0.01	0.99	160.46
P13GC-030cm	975	1,137	5.63	0.82	0.01	0.99	95.80
P13GC_036cm	775	884	5.42	0.81	0.01	0.99	85.95
P14GC	536	647	4.44	0.71	0.05	0.95	18.56
P15GC-(B)	404	502	4.78	0.80	0.03	0.97	35.12
P15GC-042cm	783	969	4.93	0.74	0.04	0.96	26.11
P15GC-051-052cm	745	885	4.97	0.75	0.03	0.97	33.28
P15GC_070cm	1,395	1,567	5.68	0.78	0.02	0.98	66.32
P15GC-090cm	794	910	5.05	0.76	0.03	0.97	36.04
P15GC-cc	1,013	1,158	6.01	0.87	0.01	0.99	125.32
P16GC-B	915	1,037	5.60	0.82	0.01	0.99	68.12
P17GC	1,151	1,307	5.81	0.82	0.01	0.99	123.35
P17GC-B-010cm	1,090	1,213	5.92	0.85	0.01	0.99	142.65
P18GC-010cm	509	637	4.41	0.71	0.03	0.97	29.24
P18GC-040cm	704	853	4.41	0.67	0.04	0.96	27.26
P18GC-070cm	498	635	4.45	0.72	0.03	0.97	29.90
P18GC-090cm	423	604	4.65	0.77	0.02	0.98	40.65
P18GC-115cm	494	601	4.51	0.73	0.03	0.97	37.31
P18GC-133cm	503	593	4.58	0.74	0.03	0.97	34.94
P18GC-160cm	507	682	4.42	0.71	0.04	0.96	24.71
P18GC-185cm	625	744	4.59	0.71	0.03	0.97	35.90
P18GC-205cm	571	659	4.47	0.70	0.04	0.96	28.31
P18GC-230cm	570	655	4.34	0.68	0.04	0.96	24.92
P18GC-245cm	538	661	4.25	0.68	0.05	0.95	20.67
P18GC-265cm	529	633	4.27	0.68	0.04	0.96	23.65
P18GC-280cm	627	692	4.55	0.71	0.03	0.97	37.22
P19GC	597	708	4.70	0.74	0.04	0.96	26.00
P20GC	742	848	4.37	0.66	0.05	0.95	18.88
P20GC-010cm	375	470	4.02	0.68	0.04	0.96	22.74
P20GC-050cm	452	584	4.29	0.70	0.04	0.96	27.74
P20GC-065cm	447	523	4.40	0.72	0.03	0.97	34.43
P20GC-080cm	422	536	4.23	0.70	0.04	0.96	24.57
P20GC-110cm	444	501	4.43	0.73	0.04	0.96	26.99
P20GC-130cm	555	647	4.18	0.66	0.07	0.93	14.63
P20GC-150cm	512	602	4.10	0.66	0.07	0.93	14.18
P20GC-175cm	417	506	4.36	0.72	0.03	0.97	30.23
P20GC-195cm	451	537	4.26	0.70	0.04	0.96	22.41
P20GC-220cm	428	499	4.15	0.68	0.06	0.94	18.06
P20GC-260cm	402	464	3.99	0.67	0.06	0.94	15.41
P20GC-280cm	391	442	4.40	0.74	0.03	0.97	35.59
P96/2GC-009cm	901	996	5.91	0.87	0.01	0.99	111.36
P69GC-008cm	950	964	5.98	0.87	0.01	0.99	174.36
P70GC-(B)	108	135	1.91	0.41	0.29	0.71	3.46
P70GC	683	727	3.92	0.60	0.08	0.92	12.76
P70GC-010cm	406	421	3.45	0.57	0.11	0.89	8.94
P70GC-030cm	194	247	2.88	0.55	0.18	0.82	5.59
P70GC-070cm	155	251	2.41	0.48	0.23	0.77	4.33
P87GC-001cm	878	999	5.92	0.87	0.01	0.99	128.82
P87GC-045cm	974	1,193	5.78	0.84	0.01	0.99	97.49
P87GC-075cm	970	1,204	5.94	0.86	0.01	0.99	161.79
P87GC-100cm	916	1,097	5.78	0.85	0.01	0.99	122.18
P87GC-150cm	644	770	4.98	0.77	0.02	0.98	44.72
P87GC-179cm	554	655	4.79	0.76	0.03	0.97	36.71
P88/2GC-top	1,131	1,374	6.18	0.88	0.00	1.00	202.13
P89/2GC-topA	1,042	1,258	6.14	0.88	0.00	1.00	204.48
P89/2GC-topB	1,111	1,345	6.07	0.87	0.01	0.99	173.94
P89/2GC-025cm	1,105	1,306	6.10	0.87	0.01	0.99	199.54
P89/2GC_042cm	1,033	1,254	5.80	0.84	0.01	0.99	121.75
P89/2GC-052cm	740	885	3.00	0.45	0.01	0.99	126.92
P89/2GC-065cm	738	901	5.36	0.81	0.01	0.99	76.83
P90-1GC	1,147	1,382	5.98	0.85	0.01	0.99	162.64
P91GC	731	833	5.30	0.80	0.02	0.98	41.22
P91GC-(B)-020cm	825	1,069	5.57	0.83	0.01	0.99	78.33
P91GC-(B)-060cm	1,026	1,068	5.28	0.76	0.02	0.98	54.76
P91GC-(B)-070cm	1,148	1,319	5.22	0.74	0.02	0.98	63.08
P91GC-(A)-105cm	831	927	4.91	0.73	0.03	0.97	39.85
P91GC-(A)-150cm	592	712	4.74	0.74	0.02	0.98	43.02
P91GC-(A)-175cm	592	652	4.45	0.70	0.03	0.97	32.26
P92GC	473	494	4.34	0.71	0.03	0.97	28.73
P92GC-(B)-030cm	234	252	3.42	0.63	0.08	0.92	13.11
P92GC-(B)-075cm	135	138	3.33	0.68	0.09	0.91	11.69
P92GC-(B)-090cm	219	223	3.95	0.73	0.04	0.96	23.93
P92GC-(A)-100cm	70	103	1.95	0.46	0.27	0.73	3.65
P92GC-(A)-130cm	53	70	1.85	0.47	0.28	0.72	3.59
P92GC-(A)-170cm	64	83	1.72	0.41	0.32	0.68	3.16
P93/2GC-008cm	915	1,064	5.79	0.85	0.01	0.99	112.86
Min	53	70	1.72	0.41	0.00	0.65	2.85
Max	1,395	1,567	6.18	0.88	0.35	1.00	204.48
Santorini	6,314	6,343	5.58	0.64	0.02	0.98	52.33
Kolumbo	6,920	6,938	6.06	0.69	0.01	0.99	68.66
Total	10,496	10,496	6.14	0.66	0.01	0.99	86.73

### 2.3. Taxonomic composition analysis

**FIGURE 3:**
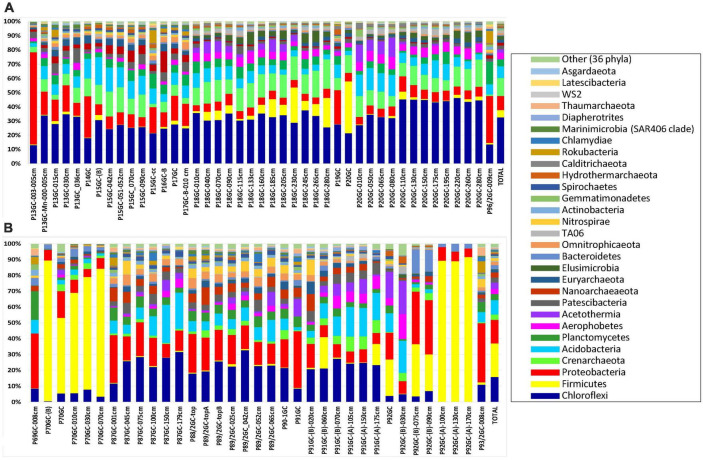
The relative abundance of major phyla in the microbial community structure on **(A)** Santorini and **(B)** Kolumbo samples.

The identified OTUs were phylogenetically assigned to 40 bacterial and 9 archaeal phyla, 14 candidate divisions and 283 bacterial and archaeal families (Figure 3 and Supplementary Table 3). Since the renaming of phyla by Oren and Garrity (2021) remains controversial among microbiologists, we use here the earlier names of phyla and in parenthesis the proposed names voted by the International Committee on Systematics of Prokaryotes which include the ending -ota. On average, the most abundant phyla in all samples were Chloroflexi (Chloroflexota) (24.62%), followed by Proteobacteria (Pseudomonadota) (11.29%), Firmicutes (Bacillota) (10.73%), Crenarchaeota (Thermoproteota) (8.55%) and Acidobacteria (Acidobacteriota) (8.07%) (Figure 3; % of total sequences). Other phyla with intermediate abundances included Planctomycetes (Planctomycetota) (4.26%), Aerophobetes (3.86%), Acetothermia (2.85%), Patescibacteria (2.69%), Nanoarchaeota (2.31%), Euryarchaeota (1.94%), Elusimicrobia (Elusimicrobiota) (1.57%), Omnitrophicaeota (1.48%), Bacteroidetes (Bacteroidota) (1.32%) etc (Figure 3 and Supplementary Table 3). Some rare phyla such as Aerophobetes, Acetothermia and Nanoarchaeota were found to be more prominent in specific samples (up to 20.20% of total sample sequences). More specifically, both Aerophobetes and Acetothermia were abundant in the sample P92GC-(B)-030cm of the hottest station 92GC of Kolumbo volcano (15.94 and 20.20%, respectively), whereas Nanoarchaeota was abundant in a single sample of Santorini caldera (i.e., P16GC-B; 14.57%). At all samples, the dominance of Proteobacteria (Pseudomonadota) was driven by the classes of Delta- (8.69%), Gamma- (5.22%), and Alphaproteobacteria (1.77%), whereas Zetaproteobacteria (0.03%), Magnetococcia (< 0.01%) and the newly characterized phylum of Epsilonbacteraeota (< 0.01%), previously known as Epsilonproteobacteria class (Waite et al., 2017), were recorded in very low abundances. The dominance of Firmicutes (Bacillota) was driven by the classes of Bacilli (13.43%), Clostridia (1.37%), and Negativicutes (0.13%), whereas within Chloroflexi (Chloroflexota), the dominant classes were Anaerolineae (17.86%) and Dehalococcoidia (15.03%). Additionally, among the most abundant classes were Bathyarchaeia (11.85%), formerly known as the Miscellaneous Crenarchaeotal group (Zhou et al., 2018), of the phylum Crenarchaeota (Ca. Bathyarchaeota phylum), Aminicenantia (10.36%) of the phylum Acidobacteria, Acetothermia (3.97%) of the phylum Acetothermia, Phycisphaerae (3.25%) of the phylum Planctomycetes (Planctomycetota), Woesearchaeia (2.60%) of the phylum Nanoarchaeota and Thermoplasmata (2.55%) of the phylum Euryarchaeota.

In Santorini caldera the prevailing phyla were Proteobacteria (Pseudomonadota) in samples P13GC-003-005cm (64.84%) and P14GC (29.42%), Firmicutes (Bacillota) in sample P20GC (36.60%) and Chloroflexi (Chloroflexota) in the rest samples (12.−44.88%). In Kolumbo volcano, predominant phyla included Firmicutes (Bacillota) (20.11%), Proteobacteria (Pseudomonadota) (14.21%), Chloroflexi (Chloroflexota) (14.76%) and Acidobacteria (8.49%). In both Santorini caldera and Kolumbo volcano, the dominance of Firmicutes (Bacillota) was driven by the family of Bacillaceae (3.28 and 12.82% of total Santorini and Kolumbo sequences, respectively) whereas the prevailing families within Chloroflexi (Chloroflexota) were Thermoflexaceae (2.92% in Santorini and 1.20% in Kolumbo sequences) and Anaerolineaceae (1.62% in Santorini and 5.29% in Kolumbo sequences). The dominance of Gammaproteobacteria was driven by the family of Pseudoalteromonadaceae in Santorini (2.17% of total Santorini sequences). However, in Kolumbo samples, the Pseudoalteromonadaceae members were almost absent accounting for less than 0.03% of total Kolumbo sequences. In both areas, Deltaproteobacteria were dominated by members of Desulfarculaceae (1.70% in Santorini and 2.50% in Kolumbo sequences) and Desulfobacteraceae (0.36% in Santorini and 1.85% in Kolumbo sequences). Other abundant families in Santorini were Pirellulaceae (0.80%) of the phylum Planctomycetes, Calditrichaceae (0.75) of the phylum Calditrichaeota, Methylomirabilaceae (0.53%) of the phylum Rokubacteria and Pseudomonadaceae (0.33%) of the phylum Proteobacteria and in Kolumbo, Clostridiaceae 1 (1.84%) of the phylum Clostridia, Weeksellaceae (1.73%) of the phylum Bacteroidetes, and Planococcaceae (1.19%) of the phylum Firmicutes.

**FIGURE 4:**
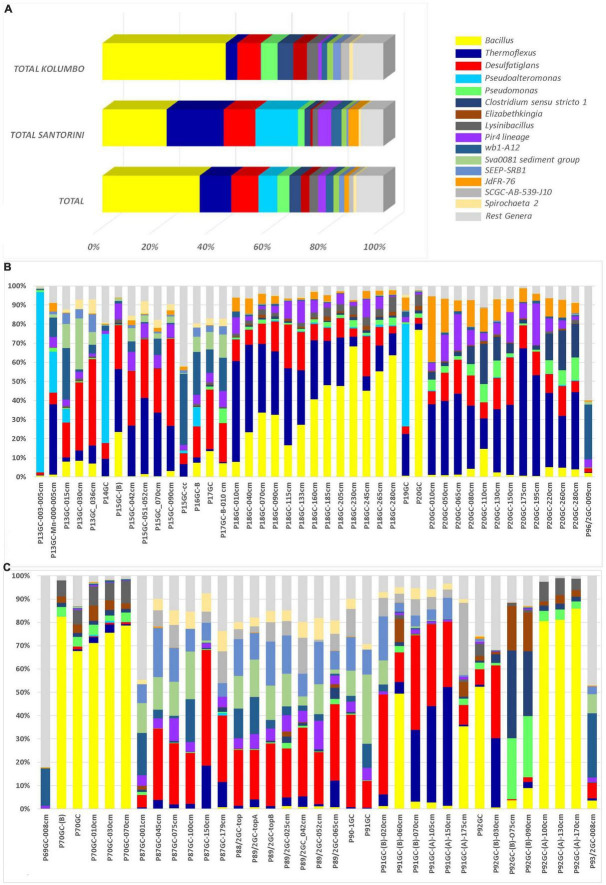
The relative abundances of the 15 major genera and candidate genera in microbial community structure **(A)** on total Kolumbo and Santorini samples and on the different sediment layers of **(B)** the Santorini and **(C)** the Kolumbo samples. Crenarchaeota (Ca. Bathyarchaeota) members were not included.

At the genus level, a total of 286 known genera and candidate genera (176 in Santorini and 223 in Kolumbo) were mostly dominated by members of *Bacillus* (3.28% in Santorini and 12.81% in Kolumbo sequences; Figure 4A), *Thermoflexus* (2.92% in Santorini and 1.20% in Kolumbo sequences; Figure 4A), *Desulfatiglans* (1.62% in Santorini and 2.45% in Kolumbo sequences; Figure 4A), *Pseudoalteromonas* (2.17% in Santorini and 0.03% in Kolumbo sequences; Figure 4A) and *Pseudomonas* (0.33% in Santorini and 1.71% in Kolumbo sequences; Figure 4A). In Santorini samples, the relative abundance of the dominant genera varied among the different sediment layers. It is interesting to note that at station 18GC, the relative abundance of *Bacillus* related sequences showed an increased trend toward the deeper sediment layers whereas the opposite trend was observed for *Thermoflexus* sequences (Figure 4B). In three samples i.e., P13GC-003-005cm, P14GC, and P19GC, the dominance of *Pseudoalteromonas* was prominent accounting for 94.29, 56.99 and 53.62% of the total sample sequences, respectively. In Kolumbo samples, we observed large differences in the distribution of the dominant genera among stations and sediment layers. In stations 87GC, 88GC, and 89/2GC, members of the genus *Desulfatiglans* (Figure 4C) were abundant whereas *Thermoflexus* appeared to dominate the sub-seafloor samples P91GC-(B)-070cm, P91GC-(A)-105cm, P91GC-(A)-150cm, P92GC-(B)-030cm, and P87GC-150cm. Members of *Pseudomonas* were found abundant in the sub-seafloor samples P92GC-(B)-075cm and P92GC-(B)-090cm, whereas *Bacillus* genus was dominant in all sediment layers of station 70GC, in the sediment layers of 60 and 175 cm of station 91GC, and in the surface and the deeper layers of station 92GC (i.e., 100 cm, 130 cm, 170 cm). Stations 69GC and 93GC showed a different genera distribution compared to the rest samples mostly dominated by members of *Woeseia* and the taxon at genus level *wb1-A12*.

### 2.4. Dominant OTUs

**FIGURE 5:**
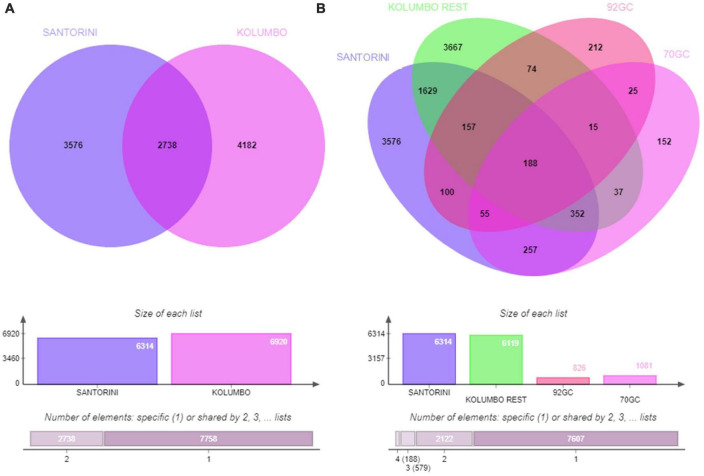
Venn diagrams showing the number of shared and unique OTUs **(A)** between Santorini and Kolumbo samples and **(B)** among Santorini samples, the hottest stations 92GC and 70GC of the Kolumbo volcano and the rest Kolumbo samples.

**FIGURE 6:**
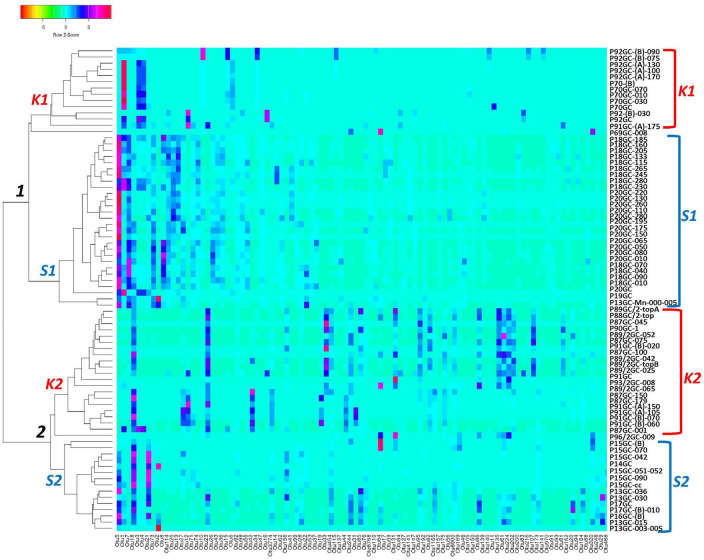
Occurrence of the major 100 OTUs clusters at 3% cutoff level (Supplementary Table 6). Cluster analysis was based on Spearman rank correlation. Color intensity signifies abundance ranging from orange (low abundance), to light blue (medium abundance) and to red (high abundance). Four clusters were formed: *K1* and *K2* (Kolumbo crater samples), *S1* and *S2* (Santorini caldera samples).

According to the Venn analysis, Santorini samples and Kolumbo samples harbor 3,576 and 4,182 unique OTUs, respectively, while sharing 2,738 OTUs (Figure 5). The hottest stations 92GC and 70GC share 500 and 852 OTUs with the Santorini samples, respectively. When comparing the hottest stations with the rest Kolumbo and Santorini samples a core of 188 OTUs was identified (Supplementary Table 4). These included the most abundant OTU which was closely related to an unidentified bacterium of the Anaerolineae class (6.64% of all sequences), and two highly abundant OTUs which were closely related to an unidentified bacterium of Aminicenantales (2.35% of all sequences), and to an unidentified archaeon of Bathyarchaeia (2.50% of all sequences). In order to detect differences among the samples, we performed heatmap analysis using the 100 most dominant OTUs. This specific analysis produced four distinct clusters according to their geographic location (Figure 6 and Supplementary Tables 5, 6). Clusters S1 and S2 consist of Santorini stations whereas clusters K1 and K2 included all Kolumbo stations. Samples from the adjacent stations 70GC and 92GC within the active area of Kolumbo volcano were grouped together in K2 cluster, whereas samples from stations located at the northern part of Santorini caldera i.e., the 18GC, 19GC and 20GC formed a well-separated cluster (S2) from the rest Santorini samples (S1 cluster). The single sample P69GC-008 located at the southern part of Santorini caldera and the sample P96/2GC-009 of Kolumbo did not group with the rest samples.

## 3. Discussion

Microbial communities obtained from the hottest station 92GC are living at the thermophilic range (> 45°C) 1 meter below the seafloor and at the hyperthermophilic range (> 75°C) at the deepest layer. Close to the seafloor, microorganisms were found to be highly diverse represented by 38 bacterial and 6 archaeal phyla including Firmicutes, Chloroflexi, Bacteroidetes, Proteobacteria, Crenarchaeota (Ca. Bathyarchaeota) etc. At the deeper layer (i.e., 170 cm), microbial communities were composed of only Firmicutes, Proteobacteria and Acetothermia. Heatmap analysis grouped the sampling stations according to their geographical location and the hottest station 92GC was placed together with the adjacent thermophilic station 70GC (Figures 1, 5). It seems that this specific area within Kolumbo volcano create a distinct niche where well adapted microorganisms can thrive. In a previous comparative analysis of microbial diversity across temperature gradients, Podar et al. (2020) have noticed that in the 67–80°C temperature range there are major shifts in the microbial communities where photosynthetic groups such as Chloroflexi, being replaced by various extreme thermophilic taxa such as Thermi, Aquificae and Crenarchaeota (Podar et al., 2020). In the case of Kolumbo station 92GC, microbial communities were dominated by members of the endospore-forming Firmicutes, Proteobacteria and Bacteroidetes compared to the rest low temperature stations where Chloroflexi was the prevailing phylum. The dominance of Firmicutes fits the Petro et al. (2017) hypothesis that microbial communities in sub-seafloor environments are driven by selection mechanisms which leave a subset of microbes from the surface to harbor favorable traits (Wörmer et al., 2019). Such traits include the formation of dormant, spore-like cells which permits microbes to face extreme environmental conditions such as intense heat and desiccation (Wörmer et al., 2019). Indeed, with a temperature at the deeper layers close to boiling point, station 92GC contains one of the most challenging environmental conditions and this is reflected on a unique microbial assembly. The dominance of spore forming cells in high temperature sediments is also confirmed by the Heuer et al. (2020) study in a subduction zone off the coast of Japan. Heuer et al. (2020) demonstrated that at temperatures above 45°C, bacterial endospores become more than 6,000 times more abundant than vegetative cells, whereas at temperatures > 100°C, acetate-degrading hyperthermophiles prevail.

Values of Chao1 and Shannon indices confirmed the high diversity of these ecosystems. Diversity values were in the same range between Santorini caldera and Kolumbo volcano sediment samples (Chao1: 70–1,567; Figure 2 and Table 1). Chao1 values were lower compared to previous estimates for microbial mat samples obtained from the polymetallic chimneys of Kolumbo volcano (Chao1: 1,309–6,973; Christakis et al., 2018), and to deep-sea hydrothermal vents of the Southwest Indian Ridge (Chao 1: 1,000–4,000; Ding et al., 2017) and higher compared to Chao1 values from other geothermal sites (i.e., Tengchong Geothermal Field, China; Chao1: 3–21; Li et al., 2015). Interestingly, the minimum Chao1 and Shannon values were calculated at the deeper layers of the hottest stations 70GC and 92GC. These results confirm previous studies in other geothermal field sites such as the Tengchong Geothermal Field in China (Li et al., 2015) and Yellowstone hot springs (Podar et al., 2020) which demonstrated that microbial communities at high temperature seem to be substantially simpler than those at the lower temperature sites.

Regarding the diversity variations with depth, in most of the stations, the Chao1 values in the deeper layers were comparable to the surface sediment samples (Figure 2 and Table 1). A recent study on global taxonomic diversity of marine sedimentary communities by Hoshino et al. (2020), showed that bacterial and archaeal richness both generally decrease with increasing depth in anoxic sediment. However, such observation was not the case for the Santorini and Kolumbo sub-seafloor microbiome even for the close to anoxic condition samples. Generally, microbial diversity decrease with depth can be ascribed to the ongoing starvation that microbes face in an ecosystem gradually depleted of available energy sources (Wörmer et al., 2019). A possible explanation for the observed diversity values at the deeper sediment layers of the present study, is that the sub-seafloor microbial life may not suffer by food limitation. Previous studies have shown that both Santorini and Kolumbo environments are characterized by a unique availability of chemical energy provided by high concentrations of several compounds such as ferrous iron (Kilias et al., 2013; Christakis et al., 2018). This is probably the main driving force in maintaining the high diversity at the subsurface of these ecosystems. This is in accordance with previous estimates by Jørgensen and Marshall (2016) who have shown that the level of diversity in sub-seafloor sediments is approximately as high as in the surface ocean.

In addition to high phylogenetic diversity, the investigated area was found to host metabolically diverse microbes. Indeed, a core of genera was found for all sediment samples and the most abundant ones included *Bacillus*, *Thermoflexus*, and *Desulfatiglans*. We further examined the ecological roles of these genera and we found that metabolically diverse bacteria are inhabiting the seafloor and sub-seafloor sediments of both Santorini caldera and Kolumbo volcano. The most abundant *Bacillus* genus is known for its ability to survive in harsh environmental conditions and its metabolic versatility, whereas the second and third most abundant *Thermoflexus* and *Desulfatiglans* genera, also known for their difficulty to be isolated in lab conditions, are associated with chemoorganotrophy, protein degradation (Thomas et al., 2021), dissimilatory sulfate reduction and aromatic hydrocarbon degradation (Jochum et al., 2018). Indeed, microorganisms of the genus *Desulfatiglans* are dissimilatory sulfate reducers that can degrade aromatic hydrocarbons in marine sediments. Little is known regarding the ecophysiology of these microbes. However, their ability to grow by utilizing aromatic organic compounds may explain their occurrence in high abundances in marine subsurface sediments (Jochum et al., 2018).

In our investigation, we examined for the first time, microbial communities inhabiting the sub-seafloor of Santorini-Kolumbo volcanic system. We uncovered the presence of diverse microbiota in this system with unique adaptation strategies including resistant forms to withstand heat stresses (e.g., endospores). It is interesting to note that in our previous investigations (Mandalakis et al., 2019; Bravakos et al., 2021) we suggested that the high concentration of reductants in Kolumbo volcano (e.g., metal enrichment) may have provided selective pressure in microorganisms to maintain resistance mechanisms including those for antibiotic resistance. Given the high and unique diversity of the sub-seafloor, we are expecting that such mechanisms can be activated in the deeper sediment layers of this volcanic system. The exploration of the deep sub-seafloor through the International Ocean Discovery Program (Expedition 398 at the Hellenic Arc Volcanic Field), will allow us to learn more about the size and the factors that shape and limit life in the deep sub-seafloor of active volcanic systems, and to reveal novel factors about the evolution of life on Earth.

## 4. Materials and methods

### 4.1. Samples collection and physicochemical analysis

Gravity corers were collected during R/V POSEIDON cruise P510 that took place in March 2017. A short (3-m long) gravity corer was used to recover sediments samples. Cores were collected along the Kolumbo line in the area of hydrothermal venting in the Kolumbo crater, and throughout the North basin of the Santorini caldera. A total of 19 corers were collected and processed for microbial community analysis. Upon recovery, the first operation was removal of the core catcher and measurement of several parameters with a portable multi-parameter probe (Lange SenSion) in the sediment (i.e., pH, Eh) and at the bottom of each corer (i.e., pH, Eh and temperature). The core sections were split longitudinally using a hand-held, power disk-saw (Fein-Multimaster), opened in two halves and subsampled for microbiology. From 10 corers only the surface layer was aseptically collected, whereas 9 corers were sliced down to different layers. Immediately after corers slicing, samples for microbiology were stored at −20°C until further analysis in the laboratory. Probe temperature measurements were made with a standard multipenetration approach using a specially redesigned heat flow probe (Modell FIELAX GmbH, Bremerhaven) consisting of a 2-m stainless steel lance attached to a modified gravity corer. Each station consisted of multiple penetrations of 15 min each for temperature measurements (Hannington et al., 2018).

### 4.2. DNA extraction, amplification and sequencing

About 1 g of material from each sample was used to extract genomic DNA from microbial communities by using the MoBio UltraClean Soil DNA isolation kit (MoBio Laboratories, Carlsbad, CA, USA) with a slightly modified protocol by the manufacturer. More specifically, the bead-beating step was replaced by a tissue lyser for at least 30 min (frequency at 30 l/s; TissueLyser II, Qiagen, Germany). Concentrations of the extracted DNA were quantified using the NanoDrop ND-1000 UV-Vis spectrophotometer (NanoDrop Technologies, USA). Then, the hypervariable V4 region of the 16S rRNA gene was amplified using the universal primers 515f (5′- GTG CCA GCMGCC GCG GTA A-3′) and 806r (5′-GGA CTA CHV GGG TWT CTA AT-3′; 25) in a PCR reaction with the KAPA HiFi HotStart DNA polymerase (1 U μl-1) (KAPA Biosystems, USA) under the following conditions: initial denaturation at 95°C for 2 min, followed by 32 cycles of 98°C for 20 s (denaturation), primer annealing at 61°C for 10 s and extension at 72°C for 15 sec, with a final extension at 72°C for 5 min. An amount of 5 μl of the PCR products was used to check amplification and intensity of the bands on a 1.5% agarose gel whereas the remaining products were purified with AMPure XP magnetic beads (Perkin-Elmer, UK). PCR products were quantified using Quant-iT PicoGreen dsDNA Assay and a TECAN Infinite F2000 Pro. The PCR negative control samples were also sequenced, in order to assess possible contamination during the library preparation. An equimolar pool of all samples was prepared and the final quantification was performed by qPCR (KAPA Library Quantification Kit−Illumina/Universal). The sequencing was performed at the premises of the Institute of Marine Biology, Biotechnology and Aquaculture (IMBBC) of HCMR in Heraklion of Crete using one run on an Illumina MiSeq with v3 chemistry for 2 × 280 cycles.

## 4.3. Data analysis

Analysis of the 16S rRNA MiSeq sequencing data was performed using the pipeline PEMA developed by IMBBC (Zafeiropoulos et al., 2020). In total, PEMA pipeline comprises 4 steps. The first one is the quality control and pre-processing of the Illumina sequencing reads using tools for the case of 16S rRNA genes such as FastQC (Andrews, 2019), Trimmomatic (Bolger et al., 2014), BayesHammer (Nikolenko et al., 2013), PANDAseq (Masella et al., 2012) and VSEARCH package (Rognes et al., 2016). The second step is the clustering of reads to operational taxonomic units (OTUs) using VSEARCH (Rognes et al., 2016). Singletons i.e., sequences with only 1 read, occurring after the (M)OTU clustering were removed from further analysis. In the third step, for the case of the 16S rRNA genes, the LCAClassifier algorithm of the CREST set of resources and tools (Lanzén et al., 2012) is used together with the Silva (Quast et al., 2013) database to assign taxonomy to the generated OTUs. In the fourth step, the PEMA’s major output is an OTU table with the assigned taxonomies and the abundances of each taxon of every sample. OTUs were defined at the species level at 97% sequence similarity. Alpha diversity which is the analysis of species diversity in a single sample, including Chao1, Shannon–Weaver and the Simpson indices was also calculated. Venn diagrams were employed to identify the shared microbial communities among the samples using the EVenn online tool (Chen et al., 2021). Cluster analysis dendrogram and heatmap were created using the freely available web server heatmapper (heatmapper.ca; Babicki et al., 2016). Cluster analysis was based on Spearman rank correlation using the first 100 most abundant OTUs which accounted for 59, 72% of the total sequences. The produced raw tag data are available through NCBI’s Sequence Read Archive under BioProject ID PRJNA898256.

## Data availability statement

The datasets presented in this study can be found in online repositories. The names of the repository/repositories and accession number(s) can be found below: https://www.ncbi.nlm.nih.gov/genbank/, PRJNA898256.

## Author contributions

PP, PN, MH, SP, SK, and AM contributed to the study design. TA, VP, EZ, and JK performed sample preparation and Illumina analysis. DL constructed the detailed bathymetry maps. PN, SW, VH, and SL collected the gravity corer samples and performed the temp/pH/Eh measurements on board the research vessel Poseidon. All authors contributed to data interpretation, article preparation, and approved the submitted version.
