# Supplementary material for: Taxonomic diversity of microbial communities in sub-seafloor hydrothermal sediments of the active Santorini-Kolumbo volcanic field
Source: Front Microbiol. 2023 Jun 29;14:1188544. doi: 10.3389/fmicb.2023.1188544 (PMC10345502; doi:10.3389/fmicb.2023.1188544)
Supplement: Supplementary file 1 [file Data_Sheet_1.zip › Supplementary Tables.pdf]

## *SUPPLEMENTARY MATERIAL*

# Taxonomic diversity of microbial communities in sub-seafloor hydrothermal sediments of the active Santorini-Kolumbo volcanic field

Paraskevi N. Polymenakou[1], Paraskevi Nomikou[2], Mark Hannington[3], Sven Petersen[3], Stephanos P. Kilias[2], Thekla I. Anastasiou[1], Vasiliki Papadimitriou[1], Eleutheria Zaka[1,4], Jon Bent Kristofersen[1], Danai Lampridou[2], Sandra Wind[5], Verena Heinath[6], Sabine Lange[3], Antonios Magoulas[1]

[1]*Hellenic Centre for Marine Research, Institute of Marine Biology, Biotechnology and Aquaculture, Gournes Pediados, P.O. Box 2214, GR 71003, Heraklion, Crete, Greece*

[2]*National and Kapodistrian University of Athens, Faculty of Geology and Geoenvironment, Panepistimioupoli Zografou, 15784 Athens, Greece*

[3]*GEOMAR, Helmhotz Centre for Ocean Research Kiel, 24148 Kiel, Germany*

[4]*University of Crete, Department of Biology, Heraklion Crete, Greece*

[5]*University of Ottawa, Department of Earth and Environmental Sciences, Ottawa, ON K1N 6N5, Canada*

[6]*University of Kiel (CAU), Institute of Geosciences, Olshausenstr. 40, D-24118 Kiel, Germany*

References of Table S5


Algora, C. Distribution of *Dehalococcoidia* in marine sediments and strategies for their enrichment. (Master of Science, Technical University of Berlin, 2016).

Bovio-Winkler, P., Cabezas, A., and Etchebehere, C. (2021). Database mining to unravel the ecology of the phylum Chloroflexi in methanogenic full scale bioreactors. *Front. Microbiol.* 11, 603234.

Bravakos, P., Mandalakis, M., Nomikou, P., Anastasiou, T. I., Kristoffersen, J. B., Stavroulaki, M., *et al.* (2021). Genomic adaptation of Pseudomonas strains to acidity and antibiotics in hydrothermal vents at Kolumbo submarine volcano, Greece. *Sci. Rep.* 11, 1336.

Dong, X., Greening, C., Rattray, J. E., Chakraborty, A., Chuvochina, M., Mayumi, D., *et al.* (2019). Metabolic potential of uncultured bacteria and archaea associated with petroleum seepage in deep-sea sediments. *Nat. Commun.* 10, 1816.

Gamba Romano, R., Gonçalves Bendia, A., Cezar Fornazier Moreira, J., Castillo Franco, D., Negrão Signori, C., Yu, T., *et al.* (2021). Bathyarchaeia occurrence in rich methane sediments from a Brazilian ría. *Estuar. Coast. Shelf Sci.* 263, 197631.

Guidi, L., Chaffron, S., Bittner, L., Eveillard, D., Larhlimi, A., Roux, S., *et al.* (2016). Plankton networks driving carbon export in the oligotrophic ocean. *Nature* 532, 465–470.

Gurunathan, R., Rathinam, A. J., Hwang, J.-S., and Dahms, H.-U. (2021). Shallow hydrothermal vent bacteria and their secondary metabolites with a particular focus on *Bacillus*. *Mar. Drugs* 19, 681.

Hao, L., Mclleoy, S. J., Kirkegaard, R. H., Karst, S. M., Eustace, W., Fernando, Y., *et al.* (2018). Novel prosthecate bacteria from the candidate phylum Acetothermia. *ISME J.* 12, 2225-2237.

Hollingsworth, A. L., Jones, D. O. B., and Young, C. R. (2021). Spatial variability of abyssal nitrifying microbes in the North-Eastern Clarion-Clipperton Zone. *Front. Mar. Sci.* 8, 663420.

Jochum, L. M., Schreiber, L., Marshall, I. P. G., Jørgensen, B. B., Schramm, A., and Kjedsen, K. U. (2018). Single-cell genomics reveals a diverse metabolic potential of uncultivated Desulfatiglans-related Deltaproteobacteria widely distributed in marine sediment. *Front. Microbiol.* 9, 1–16.

Kadnikov, V. V., Mardanov, A. V., Beletsky, A. V., Karnachuk, O. V., and Ravin, N. V. (2019). Genome of the candidate phylum *Aminicenantes* bacterium from a deep subsurface thermal aquifer revealed its fermentative saccharolytic lifestyle. *Extremophiles* 23, 189-200.

Kim, K. K., Kim, M. K., Lim, J. H., Park, H. Y., and Lee, S. T. (2005). Transfer of *Chryseobacterium meningosepticum* and *Chryseobacterium miricola* to *Elizabethkingia* gen. nov. as *Elizabethkingia meningoseptica* comb. nov. and *Elizabethkingia miricola* comb. nov. *Int. J. Syst. Evol. Microbiol.* 55, 1287–1293.

Korzhenkov, A. A., Teplyuk, A. V., Lebedinsky, A. V., Khvashchevskaya, A. A., Kopylova, Yu. G., Arakchaa, K. D., *et al.* (2018). Members of the uncultured taxon OP1 ("*Acetothermia*") predominate in the microbial community of an alkaline hot spring at East-Tuvinian Upland. *Microbiology* 87, 783–795.

Lazar, C. S., Baker, B. J., Seitz, K. W., and Teske, A. P. (2017). Genomic reconstruction of multiple lineages of uncultured benthic archaea suggests distinct biogeochemical roles and ecological niches. *ISME J.* 11, 1118-1129.

Li, J., Li, F., Yu, S., Qin, S., and Wang, G. (2013). Impacts of mariculture on the diversity of bacterial communities within intertidal sediments in the Northeast of China. *Microb. Ecol.* 66, 861-870.

Mandalakis, M., Gavriilidou, A., Polymenakou, P. N., Christakis, C. A., Nomikou, P., Medvecký, *et al.* (2019). Microbial strains isolated from $CO_2$-venting Kolumbo submarine volcano show enhanced co-tolerance to acidity and antibiotics. *Mar. Environ. Res.* 144, 102-110.

Méheust, R., Castelle, C. J., Carnevali, P. B. M., Farag, I. F., He, C., Chen, L.-X., *et al.* (2020). Groundwater *Elusimicrobia* are metabolically diverse compared to gut microbiome *Elusimicrobia* and some have a novel nitrogenase paralog. *ISME J.* 14, 2907-2922.

Nierychlo, M., Skytte Andersen, K., Xu, Y., Green, N., Jiang, C., Albertsen, M., *et al.* (2020). MiDAS 3: An ecosystem-specific reference database, taxonomy and knowledge platform for activated sludge and anaerobic digesters reveals species-level microbiome composition of activated sludge. *Water Res.* 182, 115955.

Polymenakou, P. N., Christakis, C. A., Mandalakis, M., and Oulas, A. (2015). Pyrosequencing analysis of microbial communities reveals dominant cosmopolitan phylotypes in deep-sea sediments of the eastern Mediterranean Sea. *Res. Microbiol.* 166, 448-457.

Sinkko, H., Lukkari, K., Sihvonen, L. M., Sivonen, K., Leivuori, M., Rantanen, M., *et al.* (2013). Bacteria contribute to sediment nutrient release and reflect progressed eutrophication-driven hypoxia in an organic-rich continental sea. *Plos One* 8, e67061.

Tan, S., Liu, J., Fang, Y., Hedlund, B. P., Lian, Z.-H., Huang, L.-Y., Li, J.-T., *et al.* (2019). Insights into ecological role of a new deltaproteobacterial order *Candidatus Acidulodesulfobacterales* by metagenomics and meta transcriptomics. *ISME J.* 13, 2044–2057.

Thomas, T., Evans, F. F., Schleheck, D., Mai-Prochnow, A., Burke, C., Penesyan, A., *et al.* (2008). Analysis of the *Pseudoalteromonas tunicata* genome reveals properties of a surface-associated life style in the marine environment. *PLoS ONE* 3, e3252.

Thomas, S. C., Payne, D., Tamadonfar, K. O., Seymour, C. O., Jio, J.-Y., Murugapiran, S. K., *et al.* (2021) Genomics, exometabolomics, and metabolic probing reveal conserved proteolytic metabolism of *Thermoflexus hugenholtzii* and three candidate species from China and Japan. *Front. Microbiol.* 12, 632731.

**Table S1.** Station codes, stations depth (*m*), name of samples where the sediment layer in cm is presented, geological description of the collected samples and *pH*, $E_h$ values of each sediment layer.

| ***Station*** | ***Water Column Depth (m)*** | ***Samples*** | ***pH*** | ***$E_h$ (mV)*** | ***layer of pH, $E_h$ (cm)*** | ***Lithology*** |
|---|---|---|---|---|---|---|
| ***13GC*** | ***334*** | ***P13GC-003-005cm*** | 7.19 | 147 | 5 | Fe-oxide rich mud-silt, reddish-brown, with Mn-encrustation |
| | | ***P13GC-Mn-000-005cm*** | 7.19 | 147 | 5 | Mn-crust and small feeders (0.5cm) |
| | | ***P13GC-015cm*** | 7.43 | -42 | 15 | yellow-brown silt, Fe-oxide staining, small 2mm pumice clast |
| | | ***P13GC-030cm*** | 7.69 | 152 | 30 | olive grey mud, with pumice clasts 1.5cm |
| | | ***P13GC_036cm*** | 7.05 | 182 | 35 | medium grey mud, with dark green Schlieren |
| ***14GC*** | ***331*** | ***P14GC*** | | | | core recovery, entire sample, Fe-oxide rich reddish-brown silt, with olive grey tinge, Mn-encrustation |
| ***15GC*** | ***331*** | ***P15GC-(B)*** | 6.8 | 147 | 20 | reddish brown, Fe-oxide rich silt-sand with Mn encrustation, core recovery |
| | | ***P15GC-042cm*** | 7 | 136 | 42 | reddish-brown mud-silt, Fe-oxide rich |
| | | ***P15GC-051-052cm*** | 6.96 | 97 | 52 | yellow-brown silt with olive grey tinge |
| | | ***P15GC_070cm*** | 6.99 | 86 | 74 | yellow-brown silt |
| | | ***P15GC-090cm*** | | | | coarse grained mixed volcanic hydrothermal material, very heterogenous |
| | | ***P15GC-cc*** | 6.8 | 147 | 20 | extra bag: coarse grained mixed volcanic hydrothermal material, very heterogenous |
| ***16GC-B*** | ***349*** | ***P16GC-B*** | 7.42 | 91 | 4 | Fe-oxide rich silt to fine sand, reddish-brown, with black patches |
| ***17GC*** | ***329*** | ***P17GC*** | | | | core recovery, entire sample, Fe-oxide rich silt, yellow brown, with dark and brownish-red patches |
| ***17GC-B*** | ***276*** | ***P17GC-B-010 cm*** | 7.34 | 150 | 10 | yellow-brown silt |
| ***18GC*** | ***216*** | ***P18GC-010cm*** | 6.46 | 77 | 10 | reddish brown mud (-silty), yellow brown and grey tinge |
| | | ***P18GC-040cm*** | 6.2 | 70 | 40 | reddish brown mud, with yellow brown and grey tinge |
| | | ***P18GC-070cm*** | 6.38 | 97 | 70 | strong reddish-orange brown mud (-silt) |
| | | ***P18GC-090cm*** | 6.92 | 107 | 90 | yellow brown mud with pervasive encrustation |
| | | ***P18GC-115cm*** | 6.09 | 71 | 115 | dark dusky brown, with yellow brown schlieren, silt |
| | | ***P18GC-133cm*** | 6.47 | 12 | 133 | light olive grey silt with brownish patches |
| | | ***P18GC-160cm*** | | | | mixture of yellow brown mud, dark grey black crust and greyish mud-silt |
| | | ***P18GC-185cm*** | 6.12 | 49 | 190 | dark brown mud with Fe-Mn-crust |

| | | | | | | |
|---|---|---|---|---|---|---|
| | | ***P18GC-205cm*** | 7.07 | 86 | 203 | coarse to medium grained dark brown volcaniclastic material |
| | | ***P18GC-230cm*** | 6.86 | 37 | 228 | greenish black mud |
| | | ***P18GC-245cm*** | 6.5 | 103 | 245 | dark yellow brown mud-silty, greyish schlieren |
| | | ***P18GC-265cm*** | 7.26 | 36 | 265 | dark yellow-brown silt with Mn-(Fe)-crust |
| | | ***P18GC-280cm*** | 6.82 | 165 | 280 | dark dusky brown mud to silt. With black "soft' cust |
| ***19GC*** | ***250*** | ***P19GC*** | | | | red brown mud-silt with mm-cm Fe-Mn-crusts |
| ***20GC*** | ***288*** | ***P20GC*** | 6.51 | 149 | 10 | 0-30 cm: reddish brown mud with intercalated dark grey 0.5 cm thick Fe-(Mn)-crusts ofter surrounded by a strong orange brown rim, crust is partly altered |
| | | ***P20GC-010cm*** | 6.51 | 149 | 10 | yellow to reddish brown Fe-(Mn)-oxyhydroxide crust, 9cm long, worm tubes |
| | | ***P20GC-050cm*** | 6.92 | -109 | 50 | Fe-Mb-oxyhydroxide with encrustation |
| | | ***P20GC-065cm*** | 6.26 | 145 | 65 | reddish-brown mud-silt, Fe-oxide rich |
| | | ***P20GC-080cm*** | 6.25 | 143 | 80 | Fe-oxyhydroxide, silt ranging in color from greyish, red-brown to orange-brown |
| | | ***P20GC-110cm*** | 6.1 | 89 | 110 | Fe-oxyhydroxide mud, chocolate brown with orange brown schlieren |
| | | ***P20GC-130cm*** | 5.93 | 42 | 130 | Fe-Mn-oxyhydroxide with encrustation, silt, chocolate brown |
| | | ***P20GC-150cm*** | 6.33 | 62 | 150 | Fe-Mn-oxyhydroxide silt, chocolate brown |
| | | ***P20GC-175cm*** | 6.41 | 70 | 175 | Fe-Mn-oxyhydroxide silt, orange-reddish-brown |
| | | ***P20GC-195cm*** | 6.54 | 82 | 195 | Fe-Mn-oxyhydroxide silt, cholocate brown, with orange and dark brown tinge |
| | | ***P20GC-220cm*** | 6.33 | 116 | 235 | Fe-Mn-oxyhydroxide mud, cholocate brown, with orange and dark brown tinge |
| | | ***P20GC-260cm*** | 6.32 | 86 | 265 | Fe-Mn-oxyhydroxide mud, cholocate brown, with greyish tinge |
| | | ***P20GC-280cm*** | 6.29 | 54 | 280 | Fe-Mn-oxyhydroxide mud, chocolate brown with encrustation (soft) |
| ***96-2GC*** | ***273*** | ***P96/2GC-009cm*** | 6.68 | 164 | 9 | 0-20 cm: reddish-brown mud with few small clasts, soft pieces of crust, color changes to olive brown towards the bottom |
| ***69GC*** | ***484*** | ***P69GC-008cm*** | 7.54 | 134 | 8 | Fe-oxide rich mixed layer, mud with coarse sandy material |
| ***70GC*** | | ***P70GC-(B)*** | | | | dark grey mud (silt) with clasts, mostly pumice clasts (mm-cm) |

| | | | | | | |
|---|---|---|---|---|---|---|
| | ***493*** | ***P70GC*** | 6 | -37 | 5 | 0-84 cm: clast supported homogeneous mud (silt), clasts are very heterogeneous in size ranging from mm-5 cm, mostly pumice clasts, few layered ignimbrite clasts (at 30 cm) |
| | | ***P70GC-010cm*** | | | | wet dark grey mud with clasts (mm-cm) |
| | | ***P70GC-030cm*** | 5.97 | -14 | 50 | ignimbrite clast |
| | | ***P70GC-070cm*** | 6.7 | -29 | 98 | clast supported dark grey mud |
| ***87GC*** | ***489*** | ***P87GC-001cm*** | 7.67 | 155 | 1 | orange-brown Fe-oxide rich silt |
| | | ***P87GC-045cm*** | 6.95 | -148 | 45 | homogenous clay, olive brown grey |
| | | ***P87GC-075cm*** | 7.11 | -62 | 76 | coarse sand volcaniclastic material, whitish pumice clasts and dark grey to yellow-brown in color |
| | | ***P87GC-100cm*** | 6.93 | -12 | 99 | reddish brown to orange brown layered mud (clay to silt) |
| | | ***P87GC-150cm*** | 7.16 | -98 | 150 | homogenous olive grey clay |
| | | ***P87GC-179cm*** | 7.5 | -144 | 179 | dark olive grey sandy material with dark grey schlieren, sulphide-rich |
| ***88-2GC*** | ***488*** | ***P88/2GC-top*** | | | | yellow brownish mud with orange schlieren, few small clasts |
| | | ***P89/2GC-topA*** | | | | |
| ***89-2GC*** | ***487*** | ***P89/2GC-topB*** | | | | 0-31 cm: coarse grained pumice layer, mostly light grey clasts (mm-cm), subrounded |
| | | ***P89/2GC-025cm*** | | | | 14-23 cm: yellowish brown silt |
| | | ***P89/2GC_042cm*** | | | | 37-43 cm: reddish brown mud with small clasts |
| | | ***P89/2GC-052cm*** | | | | 48-58 cm: 2 cm thick brownish mud layer followed by olive grey silt with few pumice clasts |
| | | ***P89/2GC-065cm*** | | | | 58-68 cm: dark grey silt with slight olive tinge and small clasts |
| ***90-1GC*** | ***486*** | ***P90-1GC*** | 7.67 | 155 | 30 | 0-7 cm: liquid Fe-oxide rich mud, reddish-brown in color |
| ***91GC*** | ***490*** | ***P91GC*** | | | | semiliquid olive brown mud with orange brown schlieren |
| | | ***P91GC-(B)-020cm*** | 6.86 | -74 | 20 | moderate olive brown mud (clay) |
| | | ***P91GC-(B)-060cm*** | | | | medium grained pumice clasts (<5mm), mostly light grey, few with yellowish sulphur coating |
| | | ***P91GC-(B)-070cm*** | 6.82 | -48 | 70 | olive grey clay |
| | | ***P91GC-(A)-105cm*** | 6.76 | 78 | 105 | olive grey mud (silt to fine sand material) |
| | | ***P91GC-(A)-150cm*** | 6.75 | -83 | 150 | olive grey clay |
| | | ***P91GC-(A)-175cm*** | 6.21 | -7 | 175 | fine to medium sand, dark grey material with olive tinge, dry |
| ***92GC*** | ***489*** | ***P92GC*** | | | | 0-7 cm: strong orange brown Fe-oxide rich layer |
| | | ***P92GC-(B)-030cm*** | 5.93 | 22 | 30 | moderate olive brown clay with small clasts <2mm |

| | | | | | | |
|---|---|---|---|---|---|---|
| | | ***P92GC-(B)-O75cm*** | 6.05 | 20 | 65 | medium dark grey coarse grained pumice layer, clasts up to 2 cm in size |
| | | ***P92GC-(B)-090cm*** | | | | pieces of 10 cm big light grey pumice clast, with amph. and bt. |
| | | ***P92GC-(A)-100cm*** | 6.42 | 12 | 100 | medium dark grey, dry clay |
| | | ***P92GC-(A)-130cm*** | 6.73 | 32 | 147 | medium dark grey clay with pumice clasts |
| | | ***P92GC-(A)-170cm*** | 6.86 | 64 | 170 | baked medium dark grey clay, dry |
| ***93GC*** | ***484*** | ***P93/2GC-008cm*** | 7.07 | -126 | 7 | dark grey volcaniclatic material with orange-brown schlieren and olive brown tinges, clasts <2mm |
| ***Min*** | ***216*** | | 5.93 | -148 | | |
| ***Max*** | ***493*** | | 7.69 | 182 | | |

**Table S2.** Site, stations code, stations depth (*m*), maximum recorded probe temperature, probe penetration depth at each station, and temperature measurements at the bottom of each gravity corer. Temperature probe measurements were performed in adjacent stations to gravity stations.

| Site | Station | Water Column Depth (m) | Max Probe T (°C) | Probe Penetr. (cm) | Corer length (cm) | Bottom corer T (°C) |
|---|---|---|---|---|---|---|
| Santorini Caldera | 13GC | 334 | 16.14 | 30 | 47 | 16 |
| | 14GC | 331 | 16.14 | 30 | surface sediment | 16 |
| | 15GC | 331 | 16.14 | 30 | 100 | 19 |
| | 16GC-B | 349 | 16.18 | 70 | 42 | 17 |
| | 17GC | 329 | 16.14 | 30 | surface sediment | - |
| | 17GC-B | 276 | 16.14 | 30 | 57 | 15-16 |
| | 18GC | 216 | 23.5 | 60 | 300 | 21 |
| | 19GC | 250 | 21.75 | 210 | surface sediment | - |
| | 20GC | 288 | 21.75 | 210 | 300 | 20 |
| | 96-2GC | 273 | 15.88 | - | 78 | 20 |
| Kolumbo volcano | 69GC | 484 | 16.55 | 90 | 78 | 15 |
| | **70GC** | **493** | **41.65** | **-** | **100** | **52** |
| | 87GC | 489 | 16.25 | 100 | 180 | 16 |
| | 88-2GC | 488 | - | - | 17 | 16 |
| | 89-2GC | 487 | 15.82 | 130 | 68 | 15-16 |
| | 90-1GC | 486 | 15.88 | 100 | 35 | 16 |
| | 91GC | 490 | 16.08 | 130 | 185 | 18 |
| | **92GC** | **489** | **53.57** | **100** | **190** | **99** |
| | 93GC | 484 | 20.18 | 120 | 45 | 19 |
| | Min | 216 | 15.82 | 30 | 17 | 15 |
| | Max | 493 | 53.57 | 210 | 300 | 99 |

**Supplementary Table S3.** Taxonomy results at different levels

(Excel File: Polymenakou_Supplementary_TableS3)

**Supplementary Table S4.** List of the shared OTUs among the Santorini, 92GC station, 70GC station and the rest Kolumbo samples.

(Excel File: Polymenakou_Supplementary_TableS4)

**Table S5.** Ecological roles of the most dominant OTUs

| *Taxonomy (Phylum;Class;Order;Family)* | *Genus* | *No. of Sequences* | *% all sequences* | *% Santorini sequences* | *% Kolumbo sequences* | *Ecological role* | *Reference* |
|---|---|---|---|---|---|---|---|
| *Chloroflexi;Anaerolineae;MSB-5B2* | *unclassified* | *42933* | 6.64 | 10.60 | 0.28 | uncertain due to the scarcity of isolates; dominant in solid waste and wastewater treatment plants | Bovio-Winkler et al. 2021 |
| *Firmicutes;Bacilli;Bacillales;Bacillaceae* | *Bacillus* | *31307* | *4.84* | 2.31 | 8.91 | metabolically diverse, can survive in harsh conditions | e.g. Gurunathan et al., 2021 |
| | *unclassified* | *13024* | *2.01* | 0.89 | 3.81 | | |
| | *Bacillus* | *12243* | *1.89* | 0.89 | 3.49 | | |
| *Crenarchaeota;Bathyarchaeia;unclassified;unclassified* | *unclassified* | *16322* | *2.52* | 3.91 | 0.29 | anaerobic oxidation of aliphatic and aromatic compounds, methane-rich ecosystems | Dong et al., 2019; Gamba Romano et al., 2021 |
| | *unclassified* | *9893* | *1.53* | 2.34 | 0.23 | | |
| | *unclassified* | *5384* | *0.83* | 1.33 | 0.02 | | |
| *Acidobacteria;Aminicenantia;Aminicenantales;unclassified* | *unclassified* | *15195* | *2.35* | 2.37 | 2.32 | no isolates, anaerobic organotroph capable of fermenting carbohydates and proteinaceous substrates and performing anaerobic respiration with nitrite, anaerobic oxidation of aliphatic and aromatic compounds | Dong et al., 2019; Kadnikov et al., 2019 |
| | *unclassified* | *8972* | *1.39* | 2.15 | 0.17 | | |
| | *unclassified* | *6680* | *1.03* | 0.02 | 2.66 | | |
| | *unclassified* | *4882* | *0.75* | 1.21 | 0.02 | | |
| *Proteobacteria;Gammaproteobacteria;Alteromonadales;Pseudoalteromonadaceae* | *Pseudoalteromonas* | *8697* | *1.34* | 2.16 | 0.03 | particle associated heterotrophs, carbon exporters | Guidi et al., 2016; Thomas et al., 2008 |
| *Acetothermia;Acetothermiia;unclassified;unclassified* | *unclassified* | *8192* | *1.27* | 2.03 | 0.04 | anaerobic chemoheterotrophs which obtain energy and carbon via fermantation of peptides, amino acids, and simple sugars to acetate, formate, and hydrogen | Hao et al., 2018; Korzenkov et al., 2018 |
| *Aerophobetes;unclassified;unclassified;unclassified* | *unclassified* | *8075* | *1.25* | 1.82 | 0.32 | anaerobic oxidation of aliphatic and aromatic compounds | Dong et al., 2019 |
| | *unclassified* | *5397* | *0.83* | 0.13 | 0.36 | | |
| *Chloroflexi;Dehalococcoidia;Sh765B-AG-111;unclassified* | *unclassified* | *7894* | *1.22* | 1.97 | 0.01 | putative acetogenic heterotroph | Dong et al., 2019 |
| *Elusimicrobia;29-4;unclassified;unclassified* | *unclassified* | *7381* | *1.14* | 1.85 | <0.01 | capable of heterotrophic or autotrophic lifestyles, reliant on oxygen or nitrate/nitrite-dependent respiration, or a variety of organic compounds and *Rhodobacter* nitrogen fixation (Rnf) complex-dependent acetogenesis with hydrogen and carbon dioxide as the substrates. | Méheust et al., 2020 |
| *Chloroflexi;Anaerolineae;Thermoflexales;Thermoflexaceae* | *Thermoflexus* | *7194* | *1.11* | 1.25 | 0.89 | chemoorganotrophy, protein degradation | Thomas et al., 2021 |
| | *Thermoflexus* | *2565* | *0.39* | 0.58 | 0.09 | | |
| *Firmicutes;Clostridia;Clostridiales;Clostridiaceae 1* | *Clostridium sensu stricto 1* | *5173* | *0.78* | 0.30 | 1.54 | mostly stricly anaerobic fermanting bacteria with ability to sporulate | Nierychlo et al., 2020 |
| *Chloroflexi;Anaerolineae;Anaerolineales;Anaerolineaceae* | *unclassified* | *5136* | *0.80* | 0.30 | 1.60 | anaerobic fermenters mostly found in organic-rich environments such as anaerobic methanogenic sludges or sediments. Many members benefit from hydrogenotrophy. | Sinkko et al., 2013 and references therein |
| *Firmicutes;Bacilli;Bacillales;Planococcaceae* | *Lysinibacillus* | *3961* | *0.61* | 0.27 | 1.15 | found often in intertidal sediments and contribute to nutrient cycling in marine environments through the decomposition of polymers and nondegradable OM | Li et al., 2013 |
| *Proteobacteria;Deltaproteobacteria;Sva0485;unclassified* | *unclassified* | *4802* | *0.74* | 1.19 | 0.03 | *Candidatus* Acidulodesulfobacterales; correlates strongly with ferrous iron; facultatively anaerobic autotrophs capable of nitrogen fixation; might also oxidize sulfide, depending on oxygen concentration and/or oxidation reduction potential | Tan et al., 2019 |
| *Chloroflexi;Dehalococcoidia;GIF3;unclassified* | *unclassified* | *4530* | *0.70* | 1.12 | 0.02 | potential sulphite/sulphate reducers | Algora, 2016 |
| *Bacteroidetes;Bacteroidia;Flavobacteriales;Weeksellaceae* | *Elizabethkingia* | *3990* | *0.62* | 0.11 | 1.43 | widely distributed; possesses genes of antibiotic resistance and virulence; can form a biofilm | Kim et al., 2005 |
| *Proteobacteria;Deltaproteobacteria;Desulfarculales;Desulfarculaceae* | *Desulfatiglans* | *3937* | *0.61* | 0.87 | 0.19 | dissimilatory sulfate reduction, aromatic hydrocarbons degradation | Jochum et al., 2018 |
| *Proteobacteria;Gammaproteobacteria;Pseudomonadales;Pseudomonadaceae* | *Pseudomonas* | *3235* | *0.50* | 0.15 | 1.06 | ubiquitous in the environment; cosmopolitan species; biodegradation of various organic compounds; posseses genes of antibiotic resistance | Polymenakou et al., 2015; Mandalakis et al., 2019; Bravakos et al., 2021 |
| *Euryarchaeota;Thermoplasmata;SG8-5;unclassified* | *unclassified* | *1754* | *0.27* | 0.38 | 0.10 | degradation of extracellular detrital proteins | Lazar et al., 2017 |
| *Rokubacteria;NC10;Methylomirabilales;Methylomirabilaceae* | *wb1-A12* | *1726* | *0.27* | 0.27 | 0.26 | hydrocarbon degradation, methanotroph | Hollingsworth et al., 2021 |

**Supplementary Table S6.** List of the 100 most abundant OTUs which accounted for 59,72% of the total sequences.

(Excel File: Polymenakou_Supplementary_TableS6)
